# Fluoride Release of Fresh and Aged Glass Ionomer Cements after Recharging with High-Fluoride Dentifrice

**DOI:** 10.1155/2019/9785364

**Published:** 2019-12-10

**Authors:** Fabiana Gouveia Rolim, Allan David de Araújo Lima, Islany Cardoso Lima Campos, Robson de Sousa Ferreira, Carlos da Cunha Oliveira-Júnior, Vera Lúcia Gomes Prado, Glauber Campos Vale

**Affiliations:** ^1^Restorative Dentistry Department, Federal University of Piauí, Campus Universitario Ministro Petrônio Portella, SG10, 64049-550 Teresina, PI, Brazil; ^2^Superior Education Institute of Parnaiba Valley, 64212-790 Parnaiba, PI, Brazil

## Abstract

**Objective:**

This study aimed to evaluate F release from GICs before and after recharging with F-dentifrices and after aging process.

**Methods:**

Fifteen specimens of GICs (conventional, resin modified, and high viscosity) and composite resin were stored individually in a polystyrene tube containing 2 ml of deionized water (DW), with water replacement every 24 hours. After 15 days, the specimens were treated with a dentifrice suspension (1 : 3 by volume) containing 0 *μ*g F/g (*n* = 5), 1,100 *μ*g F/g (*n* = 5), or 5,000 *μ*g F/g (*n* = 5). After 3 min, the specimens were rinsed and replaced in new tubes with 2 ml of DW. This procedure was performed 2x/day for 2 days. The readings were taken on days 1, 5, 10, and 15 before and after the treatments. A second experiment was performed, using the same specimens of the previous study that were submitted to an aging process (specimens were kept in 2 ml of DW, remaining at 37°C for 36 weeks). Readings using specific electrode for F detection were taken on days 1, 5, 10, and 15 after treatment of the samples as described above. Data were analyzed by ANOVA and Tukey's test with *α* fixed at 5%.

**Results:**

It was observed that the highest release of F for all the GICs occurred on the first day after the treatments, especially when using a high-fluoride dentifrice, with decreasing release over time. Also, although aged GICs still recharge with F treatments, the amount of F released was lower than fresh materials.

**Conclusion:**

GICs present a high F recharge and release capacity, especially in the first 24 hours and after the treatment with a high-fluoride dentifrice, even after material aging.

## 1. Introduction

The efficacy of fluoride (F) as a preventive agent for dental caries is a well-documented and recognized evidence. F decreases demineralization and activates remineralization of the enamel and dentine [1, 2]. The F mechanism of action is always the same regardless of the way of use, either by fluoridated water, fluoride dentifrices, or dental materials that release F ions to the oral cavity [3]. The presence of F in restorative materials supports the replacement of minerals on dental tissues adjacent to the restorations, minimizing the occurrence of recurrent caries lesions [4].

Thus, the presence of F in glass ionomeric cements (GICs) places these materials in focus when the clinical situation requires effective control of the oral environment, either to interrupt the caries process in the atraumatic restorations or to inhibit the occurrence of caries adjacent to restorations [5]. Indeed, two clinical randomized studies performed with school children have shown that high-viscosity glass ionomer cements used as sealants increased the interproximal F concentration [6] and provided additional protection for the tooth nearest to the sealed tooth [7], representing an effective intervention for caries prevention.

In addition to the F release property, some materials are still able to recharge with F coming from the external environment, such as that present in dentifrices or after the professional topical application of F, among others, tending to maintain the amount of F in GICs restorations [8]. Thus, the F releasing and recharge potential of GICs qualifies these materials for situations of high cariogenic challenge. However, the constant modifications in the formulation of these cements to improve their resistance can determine changes in this potential [9]. Particularly, the recharge of F in ionomers undergoing an aging process is still not sufficiently clear.

Fluoridated dentifrices are widely used and considered the most rational way to prevent dental caries [10]. Furthermore, the use of high-fluoride dentifrice (5,000 *μ*g F/g) seems to be effective in controlling root caries compared to conventional dentifrice (1,100 *μ*g F/g) in elderly patients [11] and in young patients at high risk to the development of caries [12] and has been the subject of numerous studies in recent years [13, 14]. However, no information is available about the uptake and release of F by GICs after treatment with high-fluoride dentifrice. Therefore, the aim of the present study was to evaluate the F release in glass ionomer cements (GICs) before and after recharging with high-fluoride dentifrice and after aging process.

## 2. Materials and Methods

### 2.1. Sample Preparation

**Figure 1:**
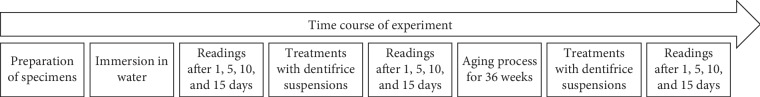
Time course of the experiment.

Sixty samples of the following materials (manipulated according to the manufacturer's instructions) were made: glass ionomer cements (conventional, resin modified, and high viscosity) and microparticulate composite resin as a negative control (Table 1). The materials were introduced in a single increment in a teflon mold (4 mm in diameter × 2 mm thick), then a polyester strip was placed on the mold and the excess material removed by pressure. The light-curing materials were photoactivated using the recommended exposure time through a polyester strip. The glass ionomer samples were protected with the manufacturer-supplied gloss to prevent water absorption during the first 24 hours. The samples were stored individually for 24 hours at 37°C in 100% humidity environment [15]. Then, each unit was conditioned in a polystyrene tube containing 2 mL of deionized water and kept in an incubator at 37°C, with water exchanged daily. After 1, 5, 10, and 15 days, the amount of F released by materials was determined (see Section 2.3). After, this first step of the study, the same samples were submitted to an aging process, which consisted in storing them in a polystyrene tube containing 2 mL of deionized water, remaining in an incubator at 37°C for 36 weeks [16]. To better understand, Figure 1 shows a time course of the experiment.

### 2.2. Treatments

To measure the recharge of materials with F, the samples were removed from the plastic tubes and the moisture excess was removed with absorbent paper and submitted to treatments by immersion in 2 ml of F-dentifrices slurries (1 : 3 by volume) containing 0 *μ*g F/g (placebo, *n* = 5), 1,100 *μ*g F/g (*n* = 5), or 5,000 *μ*g F/g (*n* = 5). The dentifrice slurries were used to simulate the saliva dilution in brushing. After 3 minutes of immersion, the specimens were rinsed for 1 minute with deionized water then returned to new containers with 2 ml of deionized water. This procedure was performed twice a day for 2 consecutive days. After this period, the samples were changed from tubes containing 2 ml of distilled water for another 15 days, with water being changed daily. F analyzes were performed on days 1, 5, 10, and 15. The same protocol described above was carried out with the aged materials.

### 2.3. Fluoride Analysis

An amount of 0.5 ml of water was removed from each tube and mixed with the same volume of TISAB II (total ionic strength adjustment buffer solution) to occur F dissociation. The F concentration was determined with a specific ion electrode (Orion 9606, Thermo Fisher Scientific, Waltham, USA) coupled to a digital potentiometer (EA-940, Thermo Fisher Scientific, Waltham, USA) using standards with 0.05 to 8 *μ*g F/mL for the standard calibration. The results were expressed in *μ*g F/mL. Additionally, the area under the curve (AUC) of the F release (*μ*g F/mL) as a function of time (days) was calculated using the program Origin 217 (One Round house Plaza, Northampton, MA, USA).

### 2.4. Statistical Analysis

The assumptions of variance equality and normal distribution of errors were verified for the response variables, which presented normal distribution. Two-way ANOVA was performed, considering the factors treatments and restorative material followed by the Tukey test. The significance level was set at 5% and statistical analysis was performed using the SAS software (version 9.0).

## 3. Results

Table 2 shows the summary of results of F release for the different materials at all experimental conditions according to the time. For all GICs materials, there was a higher release of F on the first day, tending to decrease with time, as well as a greater release when samples were treated with high-fluoride dentifrice (5,000 *μ*g F), in both fresh and aged materials. The ionomeric materials presented higher fluoride release than the composite resin.

Table 3 shows the F release for materials after the first day, according to the F-dentifrices and experimental conditions. Among the fresh GICs, there was greater fluoride release in the resin-modified GIC, regardless of the F-dentifrice treatment, which was significantly different from the resin (control group) for all treatments (*p* < 0.05). Considering the F-dentifrice treatments in fresh materials, high-fluoride dentifrice increased the F release rather than the other treatments which differed between them (*p* < 0.05).

Regarding the aged materials, it was also observed a higher F release from resin-modified GIC was compared with control, irrespective of treatment. Additionally, this GIC showed higher F release than the others GICs in 1,110 *μ*g F/g dentifrice (*p* < 0.05), which did not differ between (*p* > 0.05); while for the 5,000 *μ*g F/g treatment, both resin modified and conventional GICs enhanced F release compared to high viscosity one (*p* < 0.05). For these aged materials, high-fluoride dentifrice also increased the F release compared to other treatments (*p* < 0.05); however, no difference was observed between 0 and 1,100 *μ*g F/g treatments (*p* > 0.05).

**Figure 2:**
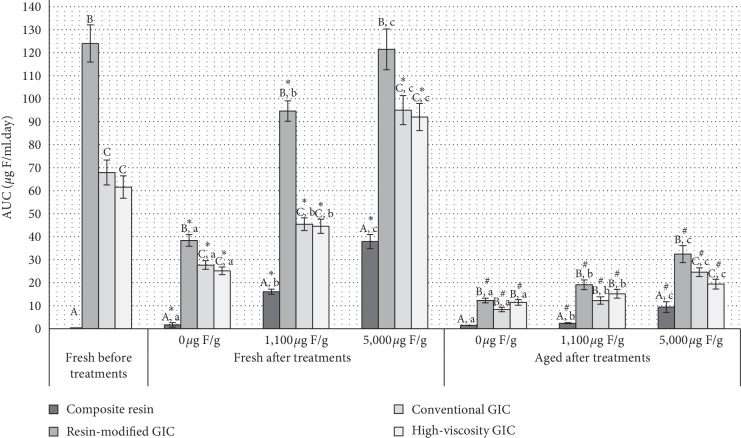
Mean of area under the curve (AUC) of the F release versus time (*μ*g F/mL.day) according to materials, treatments, and experimental conditions. Vertical bars denote standard deviations. Different uppercase letters indicate significant difference among materials in each treatment/experimental conditions and different lowercase letters indicate significant difference treatments in each material/experimental condition (*p* < 0.05). ^*∗*^indicates difference between before and after F treatments of fresh materials (*p* < 0.05). ^#^indicates difference between fresh and aged materials after each F treatments (*p* < 0.05).

**Figure 3:**
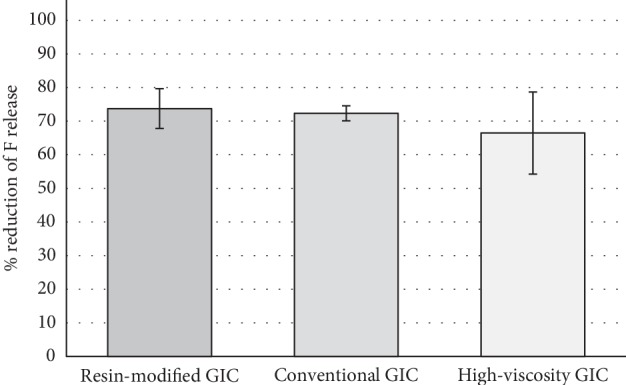
Percentage of reduction of F release according to the GICs materials after aging process (Mean ± SD, *n* = 5). No statistical difference among materials (*p* > 0.05).

The area under the curve (AUC) of F release versus time is shown in Figure 2. For fresh materials, irrespective of the treatment, resin-modified GIC had the higher F release within time (*p* < 0.05), followed by the other two GICs which did not differ (*p* > 0.05). Regarding the treatments, all of them differed, with high-fluoride dentifrice showing higher F release irrespective of the material (*p* < 0.05) and this same pattern was observed for aged materials (*p* < 0.05). However, in aged materials, GICs did not differ among them, except in 5,000 *μ*g F/g dentifrice treatment, with higher AUC for resin-modified GIC than the other two GICs material, which did not differ (*p* > 0.05). Also, it was observed a great F-release reduction (around 70%) after aging process for all GICs studied (Figure 3).

## 4. Discussion

The F release can be considered one of the main properties of GICs. The ability to recharge is also desirable since F from external sources can be absorbed by the material, thus increasing its capacity to remineralize dental structures adjacent to the restoration [4]. Although in earlier in vitro studies the F recharge of GICs from conventional dentifrices was observed [17], little was known about this property from high-fluoride dentifrice and in aged materials, which is the reason of the present research to be carried out.

High-fluoride dentifrice provides a better prevention and arrestment of carious root lesions [18, 19] in elderly and in young patients at high risk to the development of caries, and it is considered a useful therapy in caries prevention since it was able to maintain constant intraoral levels of F during 24 hours [20]. Overall, the use of the high-fluoride dentifrice leads to higher F release by the materials in the present study, showing that there is a dose-response between the F concentration of the treatment and the F release by the material (Table 2, Figure 2). This outcome may be interesting to be used by patients with high caries activity who need the oral adequation before definitive treatment.

The GICs evaluated had a higher release of F in the first 24 hours both in fresh and aged cements (Table 3, Figure 2), confirming previously obtained results [21, 22], which can be explained by the greater ionic movement in this period, which facilitates the ions release, such as F [23]. The cure reaction of GICs takes place gradually and after the first 24 hours of diffusion process, in which a small amount of F continues to be released, decreases with time [24, 25]. In fact, GICs analyzed in this study presented reduced and constant amounts of fluoride during the experimental phase as well as previous studies [23].

The aging of glass ionomer cement restorations may reduce the inhibitory effect of in situ caries since this characteristic tends to be larger immediately after restoration [26]. However, the ability to recharge glass ionomer cement is potentially beneficial in caries prevention, since it allows the formation of a slow release and storage system for the long-term dental remineralization process [5]. Although the capacity of F recharge and release had drastically decreased in aged GICs (Figure 3), it still occurs as could be observed in the present study.

The F release by the GICs occurs by dissolution and ion exchange, differently from the composite resins that occur only by ion exchange, due to the low degree of solubility of this material [17]. In the present study, composite resin (Filtek™ Z350 XT) was used as the control group. The results confirm the low release of F by the resin in relation to GICs used regardless of F-dentifrice treatment. It is also observed that the greatest release of F from the resin occurred on the first day and after treatment with 5,000 *μ*g F/g dentifrice, which could be explained by uptake of the F ion in the surface of polymeric material, since the amount of F released decreased drastically after the first day compared with the GICs.

In this experiment, samples were kept immersed in deionized water, which does not promote ionic interference. This protocol does not represent the oral environment, since the saliva components form a film on the surface of the restorative material, making F release more difficult [21]. Furthermore, it is suggested that in an acidic environment the GICs could possibly release more F because of their higher dissolution on low pH [22, 25]. Also, the literature shows that conventional GICs release higher amounts of fluoride than resin-based materials [21], different from the results of the present study. However, the unit of measurement used to present the results, the storage methods of the samples, and the different periods of the evaluations make comparative analyses difficult between studies.

In conclusion, regarding the limitations of an in vitro study, glass ionomer cements present a high F recharge and release capacity, especially in the first 24 hours and after the treatment with a high-fluoride dentifrice, even after material aging. This characteristic represents a great advantage over other restorative materials.

## Figures and Tables

**Table 1:** Restorative materials used in the study.

Materials	Composition^*∗*^	Manufacturer (batch)
Ketac Fil Plus (conventional GIG)	Powder: fluoroaluminosilicate glass, strontium, and lantanium	3M/ESPE, St. Paul, MN, USA. Color A3.
	Liquid: polycarbonic, tartaric and maleic acids, water	

Vitremer™ (resin-modified GIC)	Powder: fluoroaluminosilicate glass, redox system	3M/ESPE, St. Paul, MN, US. Color A3.
	Liquid: aqueous solution of a modified polyalkenoic acid, HEMA	

Vitro Molar® (high-viscosity GIC)	Powder: barium aluminum silicate, dehydrated polyacrylic acid, and iron oxide	DFL, Rio de Janeiro, Brazil. Color A3
	Liquid: polyacrylic acid, tartaric acid, and distilled water	

Filtek™ Z350 XT (resin)	Organic matrix: BIS-GMA, UDMA, TEGDMA, BIS-EMA, and camphorquinone	3M/ESPE, St. Paul, MN, USA. Color A2B.
	Inorganic matrix: surface-modified zircônia/sílica (3 microns or less), nonagglomerated/nonaggregated 20 nanometer surface-modified silica particles with 82% by weight (68% by volume)	

^*∗*^Manufacturers' information.

**Table 2:** Summary of results. Mean (±SD) of F (*μ*g F/ml) release from fresh and aged restorative materials before and after the treatments according to time.

Experimental condition	Material	Treatment	Time (days)			
			1	5	10	15
Fresh before treatments	Composite resin	None (*n* = 15 for each material)	0.08 (0.07)	0.04 (0.02)	0.03 (0.01)	0.03 (0.01)
	Resin-modified GIC		15.88 (4.36)	8.70 (0.98)	8.83 (0.91)	3.58 (0.51)
	Conventional GIC		8.83 (1.31)	5.09 (0.64)	4.36 (0.14)	2.22 (0.22)
	High-viscosity GIC		7.35 (1.58)	4.46 (0.40)	4.46 (0.38)	1.80 (0.17)

Fresh after treatments	Composite resin	0 *μ*g F/g (*n* = 5)	0.50 (0.16)	0.07 (0.02)	0.05 (0.02)	0.04 (0.01)
		1,100 *μ*g F/g (*n* = 5)	7.76 (1.12)	0.06 (0.02)	0.04 (0.01)	0.04 (0.01)
		5,000 *μ*g F/g (*n* = 5)	18.71 (3.29)	0.05 (0.01)	0.03 (0.01)	0.04 (0.01)
	Resin-modified GIC	0 *μ*g F/g (*n* = 5)	4.00 (0.67)	3.49 (0.51)	2.43 (0.26)	1.01 (0.14)
		1,100 *μ*g F/g (*n* = 5)	18.35 (3.76)	5.83 (1.06)	5.47 (1.03)	1.74 (0.09)
		5.000 *μ*g F/g (*n* = 5)	31.05 (3.98)	5.94 (1.23)	5.63 (1.12)	1.79 (0.11)
	Conventional GIC	0 *μ*g F/g (*n* = 5)	2.70 (0.75)	2.41 (0.86)	1.93 (0.37)	0.71 (0.15)
		1,100 *μ*g F/g (*n* = 5)	8.69 (2.06)	2.73 (0.53)	2.72 (0.65)	0.87 (0.13)
		5,000 *μ*g F/g (*n* = 5)	29.98 (4.21)	3.39 (0.42)	3.40 (0.55)	1.24 (0.10)
	High-viscosity GIC	0 *μ*g F/g (*n* = 5)	2.48 (0.33)	2.18 (0.89)	1.75 (0.24)	0.65 (0.12)
		1,100 *μ*g F/g (*n* = 5)	8.21 (2.11)	2.83 (0.74)	2.66 (0.57)	0.84 (0.12)
		5,000 *μ*g F/g	28.40 (3.85)	3.38 (0.67)	3.42 (0.79)	1.17 (0.16)

Aged after treatments	Composite resin	0 *μ*g F/g (*n* = 5)	0.42 (0.26)	0.06 (0.01)	0.05 (0.01)	0.04 (0.01)
		1,100 *μ*g F/g (*n* = 5)	0.78 (0.17)	0.04 (0.01)	0.10 (0.04)	0.04 (0.01)
		5,000 *μ*g F/g (*n* = 5)	4.45 (2.56)	0.04 (0.01)	0.05 (0.01)	0.04 (0.01)
	Resin-modified GIC	0 *μ*g F/g (*n* = 5)	3.65 (0.58)	0.51 (0.08)	0.42 (0.06)	0.23 (0.07)
		1,100 *μ*g F/g (*n* = 5)	6.62 (1.20)	0.62 (0.03)	0.46 (0.08)	0.30 (0.05)
		5,000 *μ*g F/g (*n* = 5)	12.06 (4.53)	0.90 (0.19)	0.64 (0.24)	0.43 (0.10)
	Conventional GIC	0 *μ*g F/g (*n* = 5)	2.90 (1.28)	0.27 (0.06)	0.20 (0.05)	0.13 (0.04)
		1.100 *μ*g F/g (*n* = 5)	4.66 (0.62)	0.33 (0.04)	0.22 (0.03)	0.14 (0.05)
		5.000 *μ*g F/g (*n* = 5)	10.29 (3.12)	0.44 (0.07)	0.30 (0.04)	0.21 (0.03)
	High-viscosity GIC	0 *μ*g F/g (*n* = 5)	3.42 (0.34)	0.57 (0.08)	0.32 (0.06)	0.17 (0.02)
		1.100 *μ*g F/g (*n* = 5)	4.85 (1.02)	0.63 (0.13)	0.41 (0.10)	0.24 (0.05)
		5.000 *μ*g F/g (*n* = 5)	6.04 (0.94)	0.85 (0.15)	0.53 (0.09)	0.33 (0.06)

**Table 3:** Mean (±SD) of F (*μ*g F/ml) release from fresh and aged restorative materials before and after the first day (day 1) of treatment with F-dentifrices (*n* = 5).

Experimental conditions		Materials			
		Composite resin	Resin-modified GIC	Conventional GIC	High-viscosity GIC
Before F treatments (fresh)		0.08 (0.07) A	15.88 (4.36) B	8.83 (1.31) C	7.35 (1.58) C

After F	0 *μ*g F/g	0.50 (0.39) A.a	4.00 (0.84) B.a^*∗*^	2.70 (0.83) BC.a^*∗*^	2.18 (0.42) C.a^*∗*^
treatments	1.100 *μ*g F/g	7.76 (0.70) A.b^*∗*^	18.35 (4.76) B.b	8.69 (1.41) A.b	8.31 (1.16) A.b
(fresh)	5.000 *μ*g F/g	18.71 (4.02) A.c^*∗*^	31.05 (3.35) B.c^*∗*^	29.98 (6.11) B.c^*∗*^	28.40 (9.40) AB.c^*∗*^

After F	0 *μ*g F/g	0.42 (0.26) A.a	3.65 (0.58) B.a	2.90 (1.28) B.a	3.42 (0.34) B.a
treatments	1.100 *μ*g F/g	0.78 (0.17) A.a^#^	6.62 (1.20) B.a^#^	4.66 (0.62) C.a^#^	4.85 (1.02) C.b^#^
(aged)	5.000 *μ*g F/g	4.45 (2.56) A.b^#^	12.06 (4.53) B.b^#^	10.29 (3.12) B.b^#^	6.04 (0.94) A.b^#^

Different uppercase letters indicate significant difference in rows and different lowercase letters indicate significant difference in columns in each experimental condition (*p* < 0.05). ^*∗*^indicates difference between before and after F treatments of fresh materials (*p* < 0.05). ^#^indicates difference between fresh and aged materials after each F treatments (*p* < 0.05).

## Data Availability

The data used to support the findings of this study are included within the article.
